# *In silico* analysis to explore the therapeutic potential of propolis-derived small molecules as matriptase inhibitors to suppress breast cancer growth and metastasis

**DOI:** 10.1371/journal.pone.0321687

**Published:** 2025-05-14

**Authors:** Muhammad Bilal Azmi, Han Yu, Arisha Sohail, Uzma Asif, Syed Danish Haseen Ahmed, Shamim Akhtar Qureshi, Mohnad Abdalla

**Affiliations:** 1 Computational Biochemistry Research Laboratory, Department of Biochemistry, Dow Medical College, Dow University of Health Sciences, Karachi, Pakistan; 2 Children’s Hospital Affiliated to Shandong University, Jinan, China; 3 Jinan Children’s Hospital, Jinan, China; 4 Department of Computational Biology, School of Life Sciences, Fudan University, Shanghai, China; 5 Department of Biochemistry, Medicine Program, Batterjee Medical College, Jeddah, Saudi Arabia; 6 Department of Biochemistry, University of Karachi, Karachi, Pakistan; Kwara State University, NIGERIA

## Abstract

Breast cancer is a major cause of death in women, and various drug therapies are used for its treatment. However, current therapies have many side effects and limitations. Propolis, a resinous product of bee hives, possesses a variety of biological activities, including anticancer and chemo-protective properties. The present study aimed to investigate the potential suitability of propolis-derived compounds to inhibit matriptase (MT-SP1), a potential protein target for breast cancer treatment, through comprehensive computational analysis. The MT-SP1 protein structure (PDB ID: 1EAX) was retrieved, energy-minimized, and validated. Five propolis-derived compounds with the highest binding energies to MT-SP1 were selected after virtual screening. Molecular docking of these selected ligands revealed binding energies ranging from -8.4 to -9.1 kcal/mol. Stable complex formation was validated by an additional 250 ns of molecular dynamics simulations. The HOMO-LUMO and DFT characteristics provided further evidence of the chemical reactivity and stability of these five ligands at the MT-SP1 active site. Screening of compounds for drug-likeness, pharmacokinetics (ADMET profiles), and toxicity identified two promising small molecules (PubChem IDs of ligands 72307 and 129827386) as potential drug candidates for inhibiting MT-SP1. However, experimental validation through *in vitro* or *in vivo* studies is necessary to confirm these computational findings and explore their therapeutic potential for breast cancer treatment.

## 1. Introduction

Breast cancer remains the leading form of cancer among women worldwide and continues to pose a significant public health challenge. In 2020 alone, it resulted in 685,000 deaths globally, with 2.3 million women diagnosed with the disease [1,2]. Triple-negative breast cancer (TNBC) accounts for approximately 10–15% of all breast cancer cases. It is characterized by the absence of progesterone and estrogen receptor expression, as well as the overexpression of Human Epidermal Growth Factor Receptor 2 (HER2) [3]. TNBC is more aggressive than other types of breast cancer and has fewer therapeutic options available [4]. Chemotherapy is the standard treatment and often includes drugs such as platinum-based compounds, anthracyclines, and paclitaxel. However, drug resistance is a common issue that leads to poor prognosis [5,6]. High-dose chemotherapy may be required for patients with advanced breast cancer, metastasis, or systemic recurrence, but it often causes severe adverse reactions. In light of these challenges, there is a continuous need for more effective adjuvant treatment options. Synthetic cancer treatments often target both cancerous and noncancerous dividing cells, leading to side effects such as immunosuppression, which reduces the quality of life. Additionally, natural or traditional products have demonstrated therapeutic potential due to their active medicinal constituents.

Propolis (bee glue) is a substance secreted by honey bees that fills cracks or gaps in hives and regulates humidity and temperature [7]. The composition of propolis varies based on several factors, including plant type, geographical region, beekeeping techniques, and environmental influences. Previous studies have reported that propolis exhibits strong protective effects against cancer and chemotherapy-induced damage due to its diverse array of medicinal compounds, including aliphatic compounds, aromatic acids, flavonoids, alcohols, terpenes, sugars, and esters [8,9]. These compounds promote cell death, inhibit tumor growth and abnormal gene expression, prevent blood vessels formation in tumors, and reduce DNA damage. Additionally, propolis has antioxidant, immune-boosting, and anti-inflammatory properties. Therefore, it can complement conventional and alternative cancer treatments as a supplementary therapy. Various studies have reported that propolis regulates cell cycle regulators, including cyclin D, cyclin-dependent kinases, and cyclin-dependent kinase inhibitors. This mechanism underlies the inhibition of cancer cell cycle progression, angiogenesis, and cellular growth [10]. Propolis has shown cytotoxic effects against colon, bladder, and prostate cancer cells [10–12]. Furthermore, propolis and its active compounds inhibit cancer progression by targeting Phosphoinositide 3-Kinase/Protein Kinase B (PI3K/Akt) and Mitogen-Activated Protein Kinases (MAPK) signaling pathways, leading to cell cycle arrest and apoptosis induction through both extrinsic and intrinsic pathways [13].

Recent research on genetic and molecular targets has intensified efforts to better understand the malignant progression of breast cancer. Several studies have identified key oncogenic drivers as potential therapeutic targets in metastatic breast cancer. Among these, matriptase, a type II transmembrane serine protease, plays a crucial role in the progression of TNBC [14,15]. Matriptase undergoes autoactivation in response to change in cellular microenvironment, such as pH fluctuations, oxidative stress, and other cellular factors. This process occurs through two consecutive cleavage steps after its initial production as an inactive zymogen [16–19]. Hepatocyte Growth Factor Activator Inhibitor-1 (HAI-1), a Kunitz-type transmembrane serine protease inhibitor, regulates matriptase activity by forming an inhibitory complex with it [20,21]. One of matriptase’s oncogenic effects is the activation of the Hepatocyte Growth Factor (HGF) precursor (pro-HGF), leading to the production of mature HGF. HGF, in turn, promotes cancer progression by activating multiple signaling pathways through its surface receptor, Mesenchymal-Epithelial Transition Factor (c-Met) [22–24]. For matriptase to exert its oncogenic effects, it must first be activated from its zymogens form while evading inactivation by HAI-1. Once activated, matriptase triggers the HGF/c-Met pathway, which serves as a secondary signaling cascade following Epidermal Growth Factor (EGF)-induced signaling [25]. This molecular pathway is critical for facilitating cancer cell migration, infiltration, and metastasis. Additionally, matriptase contributes to the metastasis of Michigan Cancer Foundation-7 (MCF-7) breast cancer cells via Protein Kinase C (PKC)-induced signaling. It attenuates 12-O-Tetradecanoylphorbol-13-acetate (TPA)-induced invasiveness and migration, suppresses PKC/MAPK signaling pathways, inhibits Matrix Metalloproteinase-9 (MMP 9) expression, and downregulates Nuclear Factor kappa-light-chain-enhancer of activated B cells (NF-κB)/activator protein 1 activity [26]. MT-SP1 also activates serine proteases such as urokinase-type plasminogen activator (uPA), which are crucial for angiogenesis, tumor invasion, and metastasis. Downregulating Urokinase-type Plasminogen Activator Receptor (uPAR) inhibits tumor-associated invasion by suppressing its activation [26,27]. Pawar et al. (2023) found that MT-SP1 inhibition could disrupt the PAR-2/PI3K/Akt/MMP9 axis, a pathway shared across various cancer types, including ovarian cancer. This finding suggests that MT-SP1 plays a pivotal role in tumor progression [28]. Over the past few decades, researchers have identified several MT-SP1 inhibitors, including small molecules, peptides, and antibodies [27]. However, their clinical application remains limited due to bioavailability concerns, off-target effects, and potential toxicity in vital organs [29–31]. In contrast, clinical trials of propolis-derived small molecules have demonstrated greater efficacy and improved safety in mitigating the adverse effects of cancer therapy while targeting multiple signaling pathways crucial for tumor growth and metastasis [32,33]. This study aims to explore the inhibitory potential of propolis-derived small molecules against MT-SP1 using computational methodologies. Additionally, we assess the three dimensional (3D) structures, physicochemical properties, and drug-likeness of these compounds through virtual screening, molecular docking, molecular dynamics (MD) simulations and computational pharmacokinetics screening. By analyzing the molecular interactions between propolis-derived small molecules and MT-SP1 protein, we aim to identify potential therapeutic candidates for breast cancer treatment.

## 2. Methodology

### 2.1. Target protein selection, preparation, validation and structural assessment

The structural configuration of the protein MT-SP1 (matriptase) from *Homo sapiens* (PDB ID: 1EAX) [34] was obtained from the Research Collaboratory for Structural Bioinformatics-Protein Data Bank (RCSB-PDB) website (https://www.rcsb.org/structure/1eax) [35]. This high-resolution (1.30 Å) crystal structure of matriptase provides precise and reliable structural data, making it an ideal model for docking studies and interaction analyses. The 1EAX structure includes the ligand benzamidine, a serine protease inhibitor that mimics natural substrate binding within the active site of matriptase. This ligand serves as a valuable reference for understanding the small-molecule inhibitor-binding mechanisms. The benzamidine-liganded form of matriptase presents an open and well-defined active site conformation, which is essential for modeling interactions with propolis-derived small molecules. Its presence aligns with the therapeutic objectives of this study. Additionally, the PDB deposited structure was extensively evaluated, ensuring its reliability as a control for *in silico* experiments [34].

The X-ray diffraction method was used to determine the 1EAX crystal structure, yielding R-values of 0.193 (free), 0.184 (working), and 0.184 (observed). The active site or binding site residues of MT-SP1 includes the amino acids TRP 215, SER 190, ASP 189, and HIS 57 [34]. The binding coordinates of the active site were also established to examine the amino acids interacting with the control (co-crystal ligand) [34]. For computational studies, polar hydrogens were added using the BIOVIA Discovery Studio Visualizer (https://discover.3ds.com/discovery-studio-visualizer-download), while water molecules and heteroatoms were removed [36]. Hydrogens were added to stabilize tautomeric and ionization states of the amino acid residues. The protein was initially saved in PDB format and then converted to PDBQT format using PyRx 0.8 tool (https://pyrx.sourceforge.io/) [37]. To validate the protein structure, multiple quality assessment tools were employed, including the ERRAT quality factor, Ramachandran plot, Prosa-web (https://prosa.services.came.sbg.ac.at/prosa.php) [38–41]. The Ramachandran plot was used to analyze the dihedral angle (φ versus ψ), representing potential amino acid conformations within the protein structure [40]. Additionally, the Structure Validation Server (SAVES; https://saves.mbi.ucla.edu/) was utilized to detect structural errors and analyze Z-scores, ensuring the reliability of the protein model.

### 2.2. Selection and structural optimization of propolis-derived chemical compounds

The chemical compounds reported from propolis were retrieved from PubMed literature sources [42]. The Structure Data File (SDF) format of these chemical compounds was initially obtained from the PubChem database and subsequently converted into PDB format using Open Babel software [43,44]. Using *PyRx* 0.8, the energy minimization of the selected ligands was performed with the default settings, which included: Universal Force Field, Conjugate optimization algorithm; a total of 200 steps; 1 step per update; and stopping if the energy difference was < 0.1 [37]. The chemical compounds were then converted to the PDBQT file format for further analysis. The benzamidine co-crystal complex served as a control to compare the binding energies of MT-SP1 with the selected propolis-derived compounds.

### 2.3. Molecular docking

*PyRx* is a computational platform that streamlines the virtual screening process for small-molecule library screening by automating and integrating multiple tools for molecular docking simulations [37]. In this study, *PyRx* was used to convert input files into the AutoDock Vina-compatible PDBQT file format, preparing them for docking. Docking simulations were then performed using AutoDock Vina for structure-based virtual screening of ligands within the *PyRx* 0.8 tool [37]. A 3D affinity grid box was set with dimensions of 19.5142 Å (X), 58.3964 Å (Y), and 24.7981 Å (Z) to ensure optimal coverage of the binding site. The default exhaustiveness of 8 was used in the docking simulation to generate the most optimal docked pose as an outcome.

### 2.4. Molecular dynamics simulations

Molecular dynamics (MD) simulations were conducted using Desmond 2020.1 (Schrödinger LLC) to evaluate the stability and dynamic behavior of the protein-ligand complex over a 250 ns simulation period (https://www.schrodinger.com/products/desmond) [45–50]. The 250 ns simulation duration was selected to observe time-dependent conformational changes of protein-ligand complex and to ensure the equilibration of the biosystem [51]. This duration is statistically significant, providing sufficient data points for analyzing properties such as root mean square deviation, hydrogen bond formation, and free energy estimates, allowing the biosystem to reach convergence [52]. The system was set up with the Desmond System Builder, ensuring adequate water molecule distribution throughout the simulation. The OPLS-AA (Optimized Potentials for Liquid Simulations – All Atom) force field was applied to parameterize the interactions between the protein and ligand. Solvation was performed using the TIP3P water model, with an orthorhombic simulation box that extended 10 Å beyond the protein’s surface, applying periodic boundary conditions. Counterions were added to neutralize the system, and a 0.15 M Na⁺Cl⁻ solution was included to maintain an isosmotic environment. The system underwent an equilibration phase before the production run to achieve a stable state. Simulation was conducted under physiological conditions, maintaining a temperature of 310 K and a pressure of 1.013 bar. The system was energy minimized using the steepest descent algorithm before initiating the simulation.

The SHAKE algorithm was used to constrain hydrogen bond vibrations. Long-range electrostatic interactions were computed using the Particle Mesh Ewald (PME) method. Van der Waals interactions were truncated at a 9 Å cutoff. Trajectory analysis was performed to evaluate the structural stability of the complexes, utilizing Root-Mean-Square Deviation (RMSD), Root-Mean-Square Fluctuation (RMSF), Radius of Gyration (Rg), and hydrogen bond occupancy throughout the simulation. Visualizations and graphical representations were generated to interpret atomic-level fluctuations and dynamic interactions within the simulated complexes [53,54].

### 2.5. MM-GBSA calculations

The Molecular Mechanics Generalized Born Surface Area (MM-GBSA) Gibbs free energy change (ΔGbind) calculations, along with molecular docking and MD simulations, were collectively employed to identify the most stable MT-SP1 inhibitors. This approach addressed the limitations of overestimated binding affinities in docking studies and the lack of quantitative scoring in MD simulations [55]. The MM-GBSA method was utilized to compute the binding free energy (ΔGbind). The ‘MM-GBSA’ module in Maestro 12.3 was used for these calculation, employing the OPLS-2005 force field and the VSGB 2.0 energy model as the solvent model. All other settings were applied using default parameters. The resulting binding free energies were measured in kcal/mol. The MM-GBSA binding free energies were determined using the following formula:


$$\Delta G_{bind}=G_{complex}-G_{receptor}-G_{ligand}$$

Including all interactions between the ligand and protein, the free energy of the protein-ligand complex is represented by the term G_complex_. It considers the contributions of bonded (covalent) and nonbonded interactions, including solvation effects, hydrogen bonding, van der Waals forces, and electrostatic interactions. G_receptor_ is the free energy of an unbound protein receptor that has been isolated. This signifies the energy linked to the structure of the protein and the interactions that occur within the protein, instead of with the ligand. The unbound free energy of the isolated ligand is known as the G_ligand_, which refers to the ligand structure and its internal interactions, excluding those with the protein.

### 2.6. Density functional theory (DFT) analysis

Density Functional Theory (DFT) analysis was employed to enhance the accuracy of MT-SP1 inhibitor design, providing atomic-level insights and linking molecular details [56]. DFT is widely used computational technique for studying interacting electrons and applicable to atoms, solid systems, nuclei, and quantum fluids. To conduct the analysis, we utilized the Gauss View 6.0.16 program to evaluate the most promising chemical compound, optimizing for DFT studies (Gaussian Inc., Wallingford, CT, USA, 2019) [57]. The GAUSSIAN 09 software suite was used for DFT calculations, employing the B3LYP exchange-correlation functional and the 6-31G(d,p) basis set for carbon, nitrogen, oxygen, and hydrogen atoms. The GAUSSIAN 09 platform facilitated both DFT calculations and structural interpretation of the studied molecules. Using the B3LYP/6-31G(d,p) basis set, we optimized the atomic arrangements of the chemical compound to determine its theoretical geometry. Additionally, we calculated its dipole moment (D) and single-point energies. The physicochemical parameters were also determined, including chemical hardness, chemical softness, electron affinity, electronegativity, electronic chemical energy, and the electrophilicity index. Furthermore, DFT studies were conducted to evaluate the energy gap between the lowest unoccupied molecular orbital (LUMO) and highest occupied molecular orbital (HOMO). This analysis provided a deeper understanding of chemical interactions with MT-SP1, specifically focusing on the active site of matriptase and the interaction energy of key residues.

### 2.7. Drug-likeness, toxicity, and pharmacokinetics analysis of propolis derived chemical compounds

The SwissADME web tool (http://www.swissadme.ch/) [58], OSIRIS Property Explorer (https://www.organic-chemistry.org/prog/peo/), and ADMETlab platform (http://admet.scbdd.com/home/index/) [59] were used to assess the drug-likeness, toxicity, and pharmacokinetics of the selected propolis-derived chemical compounds. These evaluation included absorption, distribution, metabolism, excretion, and toxicity (ADMET) profiles to determine their potential as drug candidates [60].

## 3. Results and discussion

### 3.1. Protein 3D model

Selecting an appropriate protein 3D model is a critical step in computational modeling, particularly in drug discovery research. In this study, we conducted a comprehensive literature review to identify a suitable protein 3D model for our proposed methodology [34]. A key consideration in protein model selection is ensuring its structural integrity and reliability for future analyses. After a thorough evaluation, we selected the protein 3D model with PDB ID: 1EAX, which represents the first binding mode of benzamidine at the S1 subsite. This model provides essential insights for the development of small-molecule inhibitors [34]. Additionally, relevant binding coordinates were explored to accurately predict and compute the binding interactions of 1EAX with the ligand/co-crystal using the AutoDock Vina program [37,61]. The affinity grid box dimensions were set as X-axis = 19.5142 Å, Y-axis = 58.3964 Å, and Z-axis = 24.7981 Å.

Since no heteroatoms, ligands, water molecules, or other undesired ions or charges were present, the downloaded PDB model was considered clean, ensuring it would not compromise the accuracy and reliability of the intended protein 3D model. The overall ERRAT-based quality factor for this model was 97.72%, indicating high structural reliability. The model also passed the Verify3D score assessment, with 83.70% of residues achieving an average 3D-1D score of ≥ 0.1. Ramachandran plot analysis showed that 89.3% of residues were in the core region, 10.7% were in the allowed zone, and no residues were found in the disallowed region. Additional structural validation matrix included bond/length angle deviation: that was 4.4, with a maximum deviation of 5.2. SAVES 3D structural G-factor analysis showed total value: 0.20, dihedral value: 0.05, and covalent value: 0.40. Planar group examination showed 95.1% of residues within the acceptable limit, and 4.9% of residues was highlighted only. We applied a previously discussed structural analysis methodology to evaluate the overall quality of the selected 3D model. The computed numerical values for total, dihedral, and covalent components confirmed the favorable structural stability of our selected protein model. Additionally, the planar group analysis further validated the model’s quality, as the majority of residues were within acceptable limits. This rigorous validation process was essential to ensure that the selected protein 3D model was suitable for molecular modeling aligned with our research objectives. The 3D structural validation results highlight the reliability and integrity of the chosen model, providing a strong foundation for further investigation into small-molecule inhibition and computational drug design strategies.

### 3.2. Experimentation for molecular docking based energetics estimation and protein-ligand interactions

Molecular docking and *in silico* analysis are powerful tools in modern drug discovery, enabling the identification of potential therapeutic effects of drug-like molecules [62]. In this study, these computational approaches helped determine the binding poses and molecular interactions of propolis-derived compounds with the MT-SP1 protein, guiding further validation of promising candidate molecules [61]. Through computational docking analysis, we screened 68 propolis-derived bioactive medicinal molecules and identified five that exhibited the strongest binding energies to MT-SP1 (target protein). Compared to the native control (co-crystal), which exhibited a binding energy of -5.5 kcal/mol, the selected propolis-derived compounds demonstrated significantly higher binding energies when docked at the active binding site of the MT-SP1. The top five ligands and their binding energies includes, sesamin (Pub Chem ID: 72307) exhibited a binding energy of -9 kcal/mol, followed by 7-epiclusianone (PubChem ID: 5471610) with -8.4 kcal/mol, (1S,4R,8S,10R)-8-benzoyl-4-(2-hydroxypropan-2-yl)-9,9-dimethyl-1,10-bis(3-methylbut-2-enyl)-3-oxatricyclo[6.3.1.02,6]dodec-2(6)-ene-7,12-dione (Pub Chem ID: 9984117) with -8.5 kcal/mol, (1S,9S,11R)-9-benzoyl-4,4,10,10-tetramethyl-1, 11-bis(3-methylbut-2-enyl)-3 oxatricyclo [7.3.1.02,7]tridec-2(7)-ene-8,13-dione (PubChem ID: 10791588) with -8.4 kcal/mol, and (s)-(+)-3-(3,4-dihydroxy-phenyl)-acrylic acid 2,2-dimethyl-8-oxo-3,4-dihydro-2h,8h-pyrano [3,2-g]-chromen-3-yl-ester (PubChem ID: 129827386) with -8.9 kcal/mol (Table 1). Based on these findings, these five propolis-derived compounds were selected for further computational experimentation.

**Table 1:** Binding energies showing the interaction of propolis-derived chemical compounds with MT-SP1 protein.

PubChem ID	Compound Name	Binding Energy (kcal/mol)
2332 (control)	Control (benzamidine)	–5.5
72307	Sesamin	–9
129827386	(s)-(+)-3-(3,4-Dihydroxy-phenyl)-acrylic acid 2,2-dimethyl-8-oxo-3,4-dihydro-2h,8h-pyrano [3,2-g]-chromen-3-yl-ester	–8.9
9984117	(1S,4R,8S,10R)-8-benzoyl-4-(2-hydroxypropan-2-yl)-9,9-dimethyl-1,10-bis(3-methylbut-2-enyl)-3-oxatricyclo[6.3.1.02,6]dodec-2(6)-ene-7,12-dione	–8.5
5471610	7-Epiclusianone	–8.4
10791588	(1S,9S,11R)-9-benzoyl-4,4,10,10-tetramethyl-1,11-bis(3-methylbut-2-enyl)-3-oxatricyclo[7.3.1.02,7]tridec-2(7)-ene-8,13-dione	–8.4
11272353	Propolone C	–8.4
10907594	aristophenone A	–8.3
122857	Germanicol	–8
637105	Nemorosone	–8
5280442	Acacetin	–8
11114020	Propolone D	–8
21721815	7-O-Prenylpinocembrin	–8
5280445	Luteolin	–7.9
5281666	Kaempferide	–7.9
5281787	Caffeic acid phenethyl ester	–7.9
4101463	5-Hydroxy-7-methoxy-2-phenylchroman-4-one	–7.8
238782	5,7-Dihydroxyflavanone	–7.8
5280681	3-Methoxyluteolin	–7.8
5281954	Tectochrysin	–7.8
6474295	Pinocembrin chalcone	–7.8
638278	Trihydroxychalcone	–7.7
5318691	Izalpinin	–7.7
5280343	Quercetin	–7.6
5281616	Galangin	–7.6
5281628	Hispidulin	–7.6
5352032	4’,5,7-Trihydroxy-3,6-dimethoxyflavone	–7.6
5377945	6-Methoxykaempferol	–7.6
259846	Lupeol	–7.5
11455669	Propolone B	–7.5
5280373	Biochanin A	–7.3
336327	Medicarpin	–7.2
44257510	Neovestitol	–7.2
92503	Vestitol	–7
637125	Communic Acid	–7
5472440	Artepillin C	–7
161692	Mecambridine	–6.9
3873459	Prenyletin	–6.9
3938139	Cearoin	–6.9
138756217	Nephrosteranic acid	–6.7
612941	12-Azabicyclo[9.2.2]pentadeca-1(14),11(15)-dien-13-one	–6.6
97030389	1-Amino-N-benzhydrylcyclopentanecarboxamide	–6.5
370	Gallic Acid	–6.4
92743	2-Amino-1-(4-nitrophenyl)propane-1,3-diol	–6.3
10228	Osthol	-6.2
689043	Caffeic Acid	–6.1
8468	Vanillic Acid	–6
2736872	2,6-Dichloro-4-(trifluoromethyl)phenylacetic acid	–6
6070438	(E)-3-(4-sulfooxyphenyl)prop-2-enoic acid	–6
637540	2-Hydroxycinnamic acid, (2E)-	–5.9
10868051	1,8-Dioxacyclotetradecane-2,9-dione	–5.9
445858	Ferulic acid	–5.8
637541	3’-Hydroxycinnamic acid	–5.8
637542	p-Coumaric acid	–5.8
10465	Heptadecanoic acid	–5.7
135	4-Hydroxybenzoic acid	–5.6
985	Palmitic Acid	–5.6
5281	Stearic Acid	–5.5
8215	Behenic Acid	–5.5
444539	Cinnamic Acid	–5.5
3026	Dibutyl Phthalate	–5.4
10467	Arachidic Acid	–5.4
243	Benzoic Acid	–5.3
6549	Linalool	–5.2
75714	Nitrocyclopentane	–4.6
5455	Thiram	–3.9
9253	Cyclopentane	–3.7

**Fig 1:**
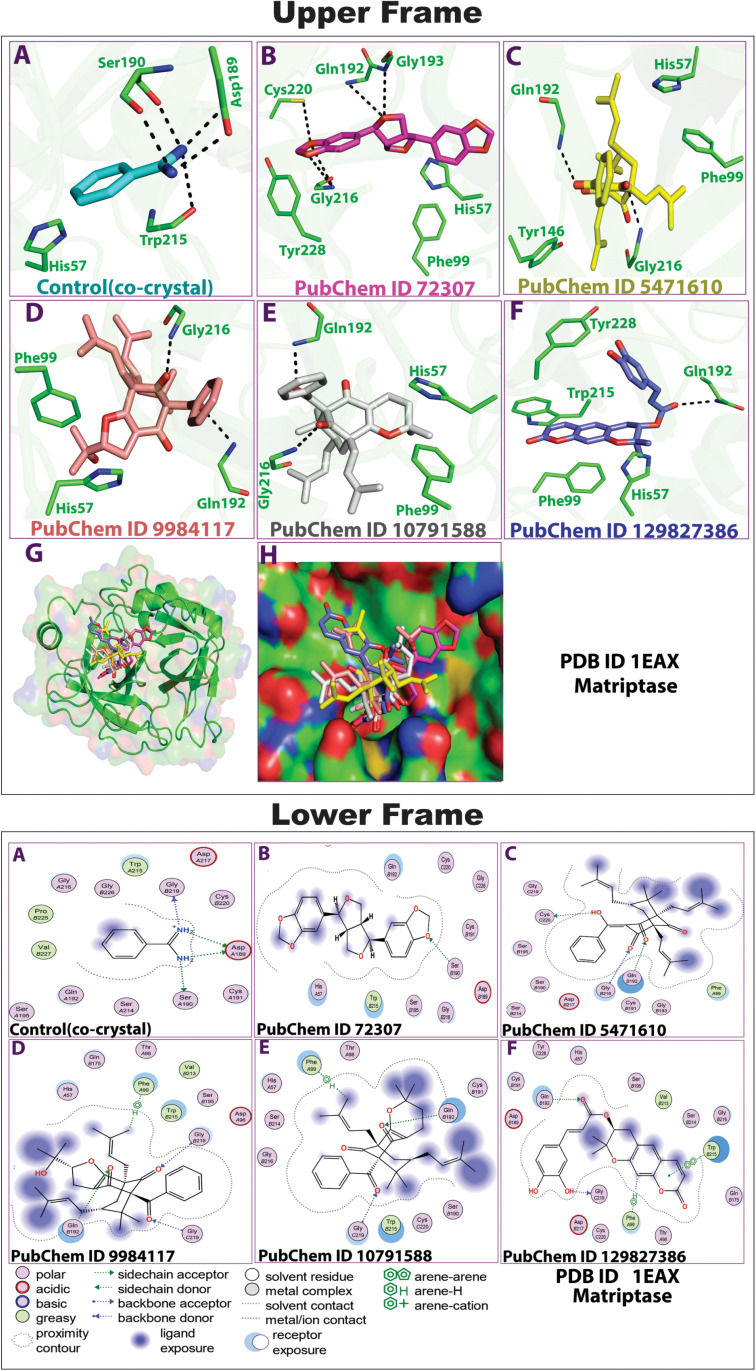
Molecular docking-based 3D (upper frame) and 2D (lower frame) binding interaction profiles of the top-ranked five propolis-derived small molecules and the control compound with key amino acid residues of MT-SP1. **(A)** Benzimidine (Control), **(B)** CID: 72307 (sesamin), **(C)** CID: 5471610 (7-epiclusianone), **(D)** CID: 9984117 [(1S,4R,8S,10R)-8-benzoyl-4-(2-hydroxypropan-2-yl)-9,9-dimethyl-1, 10-bis (3-methylbut-2-enyl)-3-oxatricyclo[6.3.1.02,6]dodec-2(6)-ene-7,12-dione)], **(E)** CID: 10791588 [(1S,9S,11R)-9-benzoyl-4,4,10,10-tetramethyl-1,11-bis(3-methylbut-2-enyl)-3-oxatricyclo[7.3.1.02,7]tridec-2(7)-ene-8,13-dione)], **(F)** CID: 129827386 [(s)-(+)-3-(3,4-Dihydroxy-phenyl)-acrylic acid 2,2-dimethyl-8-oxo-3,4-dihydro-2h,8h-pyrano [3,2-g]-chromen-3-yl-ester)].

Previous studies have identified many phytocompounds with strong binding energies; however, they often lack a comprehensive profile of protein-ligand interactions. Conventional hydrogen bonding interactions play a crucial role in biological activities, as they are essential for protein folding, molecular recognition, selectivity, and stability [62]. In addition to hydrogen bonding, the stability of the protein-ligand complexes is significantly influenced by other interactions, including hydrophobic interactions, electrostatic forces, and carbon-hydrogen bonds [62,63]. To analyze protein-ligand interactions, we used the BIOVIA Discovery Studio Visualizer to examine a subset of propolis-derived chemical compounds bound to the MT-SP1 active site (**Fig 1**). The ligand-receptor atom interactions, bond lengths, and binding energies are summarized in S1 Table. The control (co-crystal) formed conventional hydrogen bonds with ASP189, GLY219, and SER190. Ligand 72307 formed a single conventional hydrogen bond with SER190, Ligand 5471610 formed conventional hydrogen bonds with GLN192, CYS220, and GLY216, Ligand 9984117 formed pi bond with PHE99 and conventional hydrogen bonds GLN192, GLY216, and GLY219. Ligand 10791588, formed a single pi (π) bond with PHE99 and conventional hydrogen bonds with GLN192 and GLY219 and Ligand 129827386 formed conventional hydrogen bonds with GLN 192 and GLY219, while a single bond with PHE99 and pi-pi bond with TRP 215 (S1 Table).

The hydrogen bond interaction between SER190 (a key amino acid in the MT-SP1 catalytic triad) and ligand 72307 was comparable to that of the control (benzamidine) and contributed to direct inhibition. ASP189 plays a crucial role in substrate specificity and active site stability, as it interacts with the control ligand (benzamidine). However, none of the propolis compounds formed interactions with ASP189. Despite this, their ability to bind to neighboring GLY219 and GLN192 suggests a potential influence on substrate-binding site and overall enzyme stability. Notably, propolis compounds 5471610, 9984117, 10791588, and 129827386 formed hydrogen bonds with GLY216, GLY219, and GLN192, respectively, residues essential for substrate recognition and stabilization. These interactions suggest a potential blockage of substrate access, thereby inhibiting MT-SP1 activity. Additionally, the pi (π) and pi-pi (π-π) interactions between PHE99, TRP215, and compounds 9984117, 10791588, and 129827386 may enhance ligand-binding energy and stability within the active site, likely increasing their inhibitory effects. The various interactions observed between MT-SP1 and propolis compounds highlight multiple key residues within the active site that could contribute to effective MT-SP1 inhibition. Investigating protein-ligand interactions is crucial for drug discovery, as it facilitates the identification, optimization, and prioritization of promising drug candidates [64,65]. This approach enhances efficiency, cost-effectiveness, and precision, while providing deeper insights into binding mechanisms [56,64,65].

### 3.3. MD simulation

**Fig 2:**
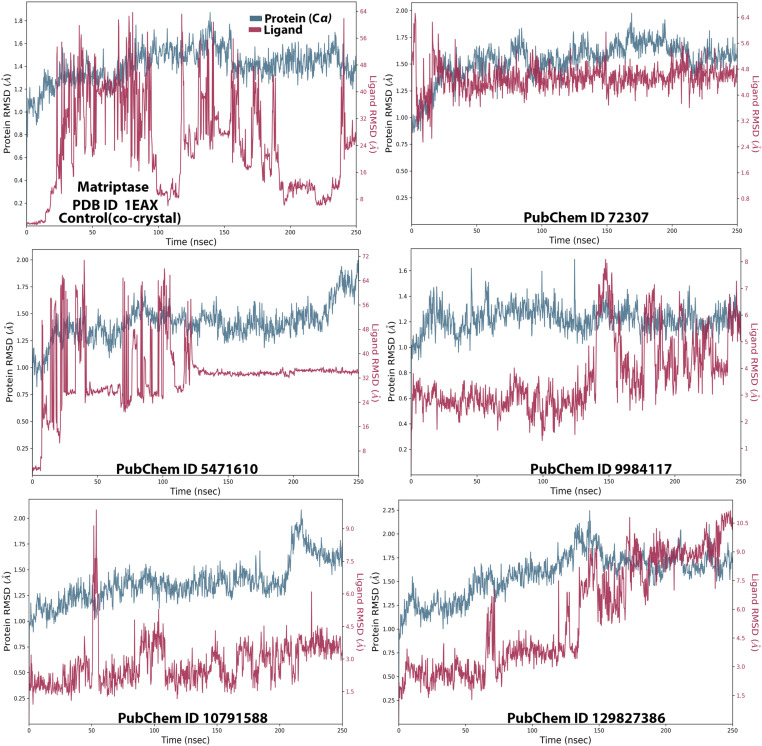
Time-dependent RMSD analysis of MT-SP1 in complex with the top-ranked five propolis-derived small molecules and the control compound. (Control/Co-crystal) Benzimidine, (PubChem ID 72307) sesamin, (PubChem ID 5471610) 7-epiclusianone, (PubChem ID 9984117) 1S,4R,8S,10R)-8-benzoyl-4-(2-hydroxypropan-2-yl)-9,9-dimethyl-1,10-bis(3-methylbut-2-enyl)-3-oxatricyclo[6.3.1.02,6]dodec-2(6)-ene-7,12-dione, (PubChem ID 10791588) 1S,9S,11R)-9-benzoyl-4,4,10,10-tetramethyl-1,11-bis(3-methylbut-2-enyl)-3-oxatricyclo[7.3.1.02,7]tridec-2(7)-ene-8,13-dione, (PubChem ID 129827386) (s)-(+)-3-(3,4-Dihydroxy-phenyl)-acrylic acid 2,2-dimethyl-8-oxo-3,4-dihydro-2h,8h-pyrano [3,2-g]-chromen-3-yl-ester.

**Fig 3:**
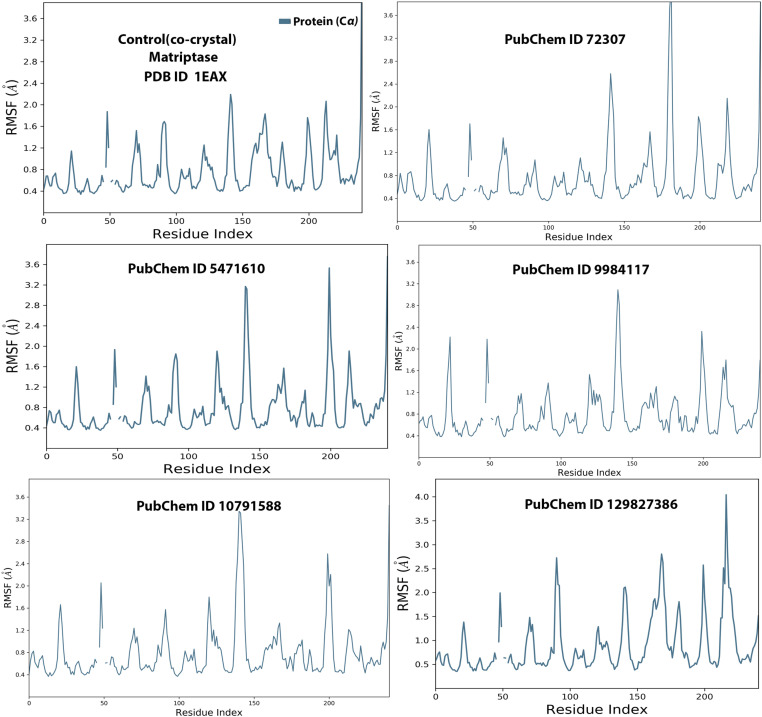
The root mean square fluctuation (RMSF) plot of MT-SP1 with the top-ranked five propolis-derived small molecules and the control compound based on Cα atoms of receptor protein. (Control/Co-crystal) Benzimidine, (PubChem ID 72307) sesamin, (PubChem ID 5471610) 7-epiclusianone, (PubChem ID 9984117) 1S,4R,8S,10R)-8-benzoyl-4-(2-hydroxypropan-2-yl)-9,9-dimethyl-1,10-bis(3-methylbut-2-enyl)-3-oxatricyclo[6.3.1.02,6] dodec-2(6)-ene-7,12-dione, (PubChem ID 10791588) 1S,9S,11R)-9-benzoyl-4,4,10,10-tetramethyl-1,11-bis(3-methylbut-2-enyl)-3-oxatricyclo[7.3.1.02,7] tridec-2(7)-ene-8,13-dione, (PubChem ID 129827386) (s)-(+)-3-(3,4-Dihydroxy-phenyl)-acrylic acid 2,2-dimethyl-8-oxo-3,4-dihydro-2h,8h-pyrano [3,2-g]-chromen-3-yl-ester.

MD simulation experiments are crucial for the development and validation of novel therapeutic agents/drugs, as they provide insights into the molecular behavior and dynamic interactions of ligands or chemical molecules with target proteins or biomacromolecules in biological systems [53,66]. The MD simulation-based approach utilizes classical physical principles to study the molecular behavior of atoms and molecules over time. By tracing the resulting trajectories of molecules, researchers can observe interactions, structural motions, and conformational changes, uncovering key properties and mechanisms of protein-ligand interactions in molecular systems [64,66]. In our computational study, the MD simulation approach was performed to analyze the optimal chemical compounds (selected propolis-derived small molecules based on docking scores) with MT-SP1 (protein target) over 250 ns. The simulated Desmond trajectories were analyzed to compute the root-mean-square deviation (RMSD) and root-mean-square fluctuation (RMSF), providing the insights into the time-dependent variation of the protein-ligand complexes. The RMSD values for the C-alpha atoms in all selected ligands bound to MT-SP1 fluctuated over the 250 ns simulation period, as shown in **Fig 2**. The RMSD plots for all top-ranked propolis–derived molecules with MT-SP1 exhibited satisfactory stabilization, ranging from 1.0 to 2.25 Å, indicating that the ligand-protein complexes remained stable throughout the simulation [53,67]. When comparing ligand fit to Protein RMSD with the control (co-crystal), compounds 72307, 9984117, 129827386, and 10791588 demonstrated greater stability with MT-SP1 during the 250 ns simulation. The RMSD for ligands 9984117 and 129827386 remained constant up to 140 ns, followed by a minor fluctuation between 4 and 5 Å, likely due to ligand mode variation, which quickly stabilized. Compound 10791588 exhibited a sudden RMSD fluctuation at 50 ns, but eventually stabilized, maintaining equilibrium for the remainder of the simulation (**Fig 2**). These findings suggest the potential inhibitory activity of propolis-derived small molecules against MT-SP1, paving the way for further experimental validation of their therapeutic efficacy in breast cancer models. To analyze protein flexibility and ligand stability, RMSF-dependent residue-specific variations were examined (**Fig 3**). The RMSF values (peaks) indicated that most residues with lower RMSF were located at the protein’s binding site, suggesting effective engagement of propolis-derived small molecules with critical MT-SP1 residues. To gain a comprehensive understanding, the MD simulation was extended to assess root mean square fluctuations (RMSFs), secondary structural elements (SSEs), and ligand-protein residues interactions. RMSF variations help identify flexible protein regions, which can reveal potential conformational changes upon ligand binding [64]. Secondary structural element (SSE) distribution provides insights into protein stability and structural integrity upon ligand binding [68]. The RMSF values of most MT-SP1 (co-crystal) amino acids remained <2.00 Å during the 250 ns simulation. In the 72307 ligand-protein analysis, RMSF values remained low (*i.e.,* 0.8–2.5 Å), except for a few residues (positions 190–200 and at the terminal end of the protein chain). Similarly, for ligands 9984117, 5471610, 10791588, and 129827386, a minor increase in the peak for each ligand for residues found between 140 and 150 positions for ligands 5471610 and 10791588, at 200 positions for ligand 5471610, and between 210 and 220 for 129827386 was observed. In contrast, the RMSF plot analysis of MT-SP1 with the co-crystal showed a stable pattern, i.e., 0.4–2.0 Å (**Fig 3**). Residues with higher RMSF values indicated regions of MT-SP1 with greater atomic displacement, possibly corresponding to loop regions or arears near the active site. These regions reflect the conformational adaptability of MT-SP1 in response to ligand binding. Notably, high fluctuations were observed in residues GLN192, SER190, and GLY219, which were key interacting residues in docking studies. This suggests that the active site of MT-SP1 exhibits a dynamic environment, potentially influencing ligand specificity and binding energy. Matriptase (MT-SP1) require a balance between flexibility and rigidity for optimal function. Residues with high RMSF values may participate in allosteric regulation, affecting overall enzymatic activity. If ligands induce excessive flexibility in the catalytic triad or substrate-specific sites, enzyme activity may be disrupted, indicating their potential role in anti-cancer therapy. These findings provide valuable insights for designing more effective inhibitors by targeting flexible regions to enhance inhibitory potency. Conversely, fluctuations in some ligands may indicate weaker binding interactions, highlighting the need for structural optimization to enhance active site rigidity. Overall, RMSD and RMSF analyses confirm that the docked MT-SP1/ligand complexes exhibited stable interaction pattern with minimal variation (Fig 2 and 3).

**Fig 4:**
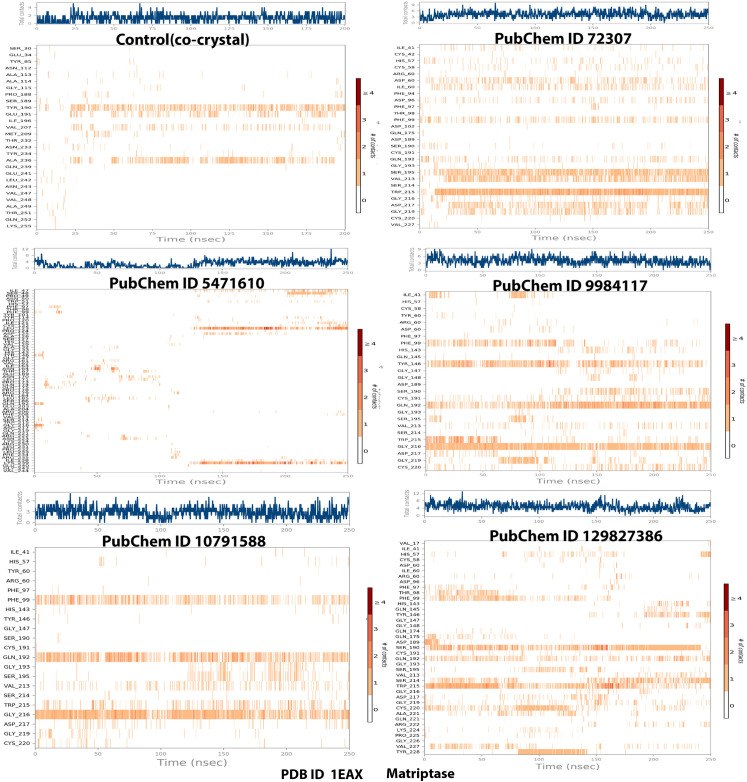
Interaction timeline of MT-SP1 with the top-ranked five propolis-derived small molecules and the control compound, where color intensity represents contact frequency throughout the simulation. (Control/Co-crystal) Benzimidine, (PubChem ID 72307) sesamin, (PubChem ID 5471610) 7-epiclusianone, (PubChem ID 9984117) 1S,4R,8S,10R)-8-benzoyl-4-(2-hydroxypropan-2-yl)-9,9-dimethyl-1,10-bis(3-methylbut-2-enyl)-3-oxatricyclo [6.3.1.02,6]dodec-2(6)-ene-7,12-dione, (PubChem ID 10791588) 1S,9S,11R)-9-benzoyl-4,4,10,10-tetramethyl-1,11-bis(3-methylbut-2-enyl)-3-oxatricyclo [7.3.1.02,7]tridec-2(7)-ene-8,13-dione, (PubChem ID 129827386) (s)-(+)-3-(3,4-Dihydroxy-phenyl)-acrylic acid 2,2-dimethyl-8-oxo-3,4-dihydro-2h,8h-pyrano [3,2-g]-chromen-3-yl-ester.

**Fig 5:**
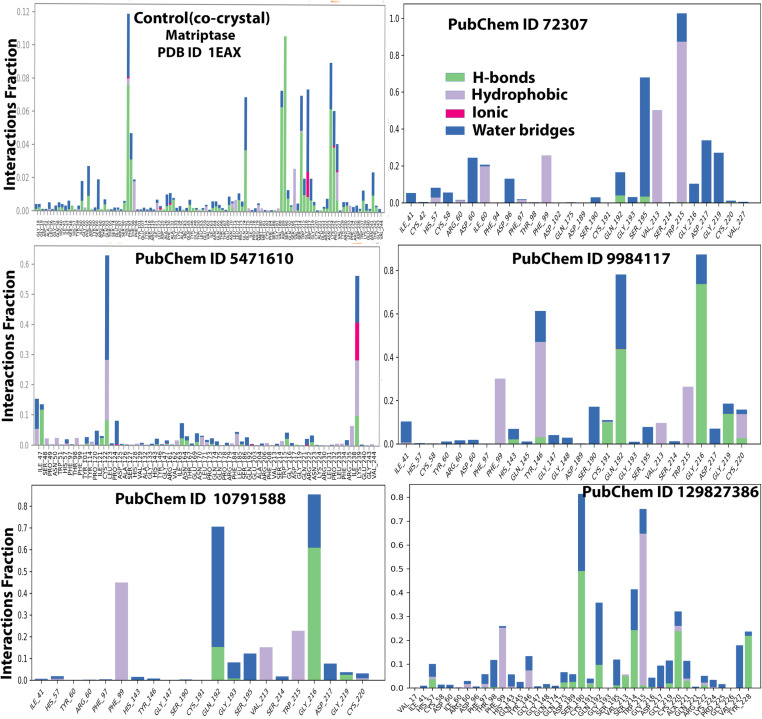
Molecular interactions between MT-SP1 with top-ranked five propolis-derived small molecules and the control compound, illustrating distinct bonding patterns and key stabilizing forces. (Control/Co-crystal) Benzimidine, (PubChem ID 72307) sesamin, (PubChem ID 5471610) 7-epiclusianone, (PubChem ID 9984117) 1S,4R,8S,10R)-8-benzoyl-4-(2-hydroxypropan-2-yl)-9,9-dimethyl-1,10-bis(3-methylbut-2-enyl)-3-oxatricyclo[6.3.1.02,6] dodec-2(6)-ene-7,12-dione, (PubChem ID 10791588) 1S,9S,11R)-9-benzoyl-4,4,10,10-tetramethyl-1,11-bis(3-methylbut-2-enyl)-3-oxatricyclo[7.3.1.02,7] tridec-2(7)-ene-8,13-dione, (PubChem ID 129827386) (s)-(+)-3-(3,4-Dihydroxy-phenyl)-acrylic acid 2,2-dimethyl-8-oxo-3,4-dihydro-2h,8h-pyrano [3,2-g]-chromen-3-yl-ester.

**Fig 6:**
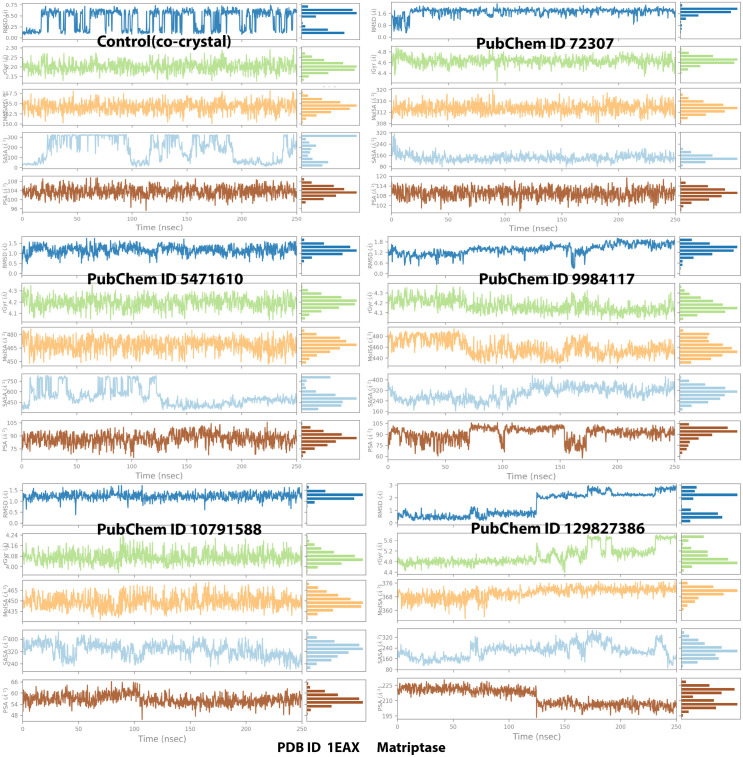
Computation of ligand properties for the top-ranked propolis-derived small molecules and the control compound in complex with MT-SP1 during MD simulation analysis. (Control/Co-crystal) Benzimidine, (PubChem ID 72307) sesamin, (PubChem ID 5471610) 7-epiclusianone, (PubChem ID 9984117) 1S,4R,8S,10R)-8-benzoyl-4-(2-hydroxypropan-2-yl)-9,9-dimethyl-1,10-bis(3-methylbut-2-enyl)-3-oxatricyclo[6.3.1.02,6]dodec-2(6)-ene-7,12-dione, (PubChem ID 10791588) 1S,9S,11R)-9-benzoyl-4,4,10,10-tetramethyl-1,11-bis(3-methylbut-2-enyl)-3-oxatricyclo[7.3.1.02,7]tridec-2(7)-ene-8,13-dione, (PubChem ID 129827386) (s)-(+)-3-(3,4-Dihydroxy-phenyl)-acrylic acid 2,2-dimethyl-8-oxo-3,4-dihydro-2h,8h-pyrano [3,2-g]-chromen-3-yl-ester.

**Fig 4** illustrates the interaction timeline between the top-ranked selected ligands and the co-crystal bound to the MT-SP1 protein. The top panel presents the total number of distinct contacts formed between the protein and ligand over time, providing an overview of interaction dynamics (represented by the blue line). The bottom panel highlights the specific residues engaged with the ligand in each trajectory frame, where darker shades indicate multiple contacts with the same residue. The scale on the right side of the plot shows that certain residues establish multiple specific contacts with the ligand, represented by the darker orange shade (**Fig 4**). Throughout the simulation, protein-ligand interactions were categorized into four types: hydrogen bonds, hydrophobic interactions, ionic interactions, and water bridge interactions. **Fig 5** displays the specific residues involved in the interactions between the five protein-ligand complexes (ionic, hydrophobic, water bridges, and hydrogen bonds). **Fig 5** depicts the specific residues involved in these interactions across the five protein-ligand complexes. During the simulation, ligand 72307 engaged with active site residues, particularly HIS57, SER190, and SER195, which are involved in catalytic interactions. Additionally, SER195 and TRP215 formed hydrogen bonds, hydrophobic interactions, and water bridges with the ligand 72307. The interaction fraction, displayed on the Y-axis of **Fig 5**, indicates the duration of each interaction type over the 250 ns simulation period. HIS57 exhibited an interaction fraction of 0.1, with 5% attributed to water bridges and 5% to hydrophobic bonds. Similarly, SER190 demonstrated a 1% interaction fraction for water bridges. For ligand 9984117, interaction with SER190 and SER 195 were observed, with a 20% interaction fraction for water bridges. Additionally, ligand 9984117 formed multiple conventional hydrogen bonds with CYS191, GLN192, GLY216, and GLY219 throughout the simulation. Ligand 129827386 interacted with active site residues SER190 and HIS57, showing an interaction proportion of 30% for water bridges and 50% for hydrogen bonds with SER190. HIS57 exhibited a 5% interaction fraction for hydrogen bonds and 5% for water bridges. Furthermore, ligand 129827386 established water bridges with SER195 and formed a significant number of conventional hydrogen bonds with GLN175, ASP189, CYS191, GLN192, VAL213, SER214, GLY219, CYS220, ALA221, ARG222, and TYR228. Ligand 10791588 demonstrated a small interaction fraction of 1% for water bridges but exhibited significant conventional hydrogen bonding with GLN192, GLY216, and GLY219 (Figs 4 and 5). Fig 6 presents the ligand properties of all selected top-ranked ligands and the control (co-crystal) complexed with the MT-SP1 protein. Typically, ligand RMSD is measured relative to the reference conformation (with time t=0 serving as the initial frame). Additionally, parameters such as the Radius of Gyration (rGyr), Molecular Surface Area (MolSA), solvent-accessible surface area (SASA), and Polar Surface Area (PSA) were computed (**Fig. 6**). Benzamidine, serving as a control, exhibited high stability, enabling comparison with the selected ligands. The structural stability and solvent interaction profiles of ligands 5471610 and 129827386 indicated superior solvation potential compared to benzamidine, suggesting strong binding affinity to the MT-SP1 protein. Ligand 10791588 demonstrated stability comparable to benzamidine, whereas, ligands 72307 and 9984117 displayed lower solvent exposure and moderate stability. The chromene and dimethyl moieties in compound 129827386 were well suited for hydrophobic interactions with MT-SP1’s hydrophobic pockets. High SASA and PSA values suggested enhanced polar interactions due to hydroxyl groups and ester linkages, which likely formed hydrogen bonds with the MT-SP1 protein, contributing to inhibitor stability and bioavailability. Ligand 5471610, with its tricyclic core and aromatic substituent, effectively occupied the hydrophobic pockets of MT-SP1’s active site. Its high SASA and PSA values facilitated ligand desolvation and binding, likely through ketone and ester groups, enhancing hydrogen bonding with the polar residues. However, excessive steric bulk might hinder the entry of these compounds into MT-SP1’s deeper binding pockets. Optimizing their structures by replacing bulky groups with smaller moieties and adjusting hydroxyl group orientation may improve binding. An *in silico* structure-activity relationship (SAR) analysis confirmed that these compounds contain functional elements influencing their inhibitory activity against MT-SP1, suggesting their potential role in breast cancer management.

MD simulation analysis revealed that all five propolis-derived small molecules were more stable than the control (co-crystal complex). During the 250 ns simulation, the selected compounds demonstrated stability at the active site (**Figs 2 and 3**). However, compared to the selected compounds, the co-crystal complex formed a greater number of hydrogen bonds with the active site residues throughout the simulation (**Fig 5**). Since the propolis-derived small molecules and the co-crystal complex exhibited comparable results, determining which complex was more stable remained challenging. Therefore, additional protein-ligand complex evaluations were conducted to gain further insights.

### 3.4. Principal component analysis

**Fig 7:**
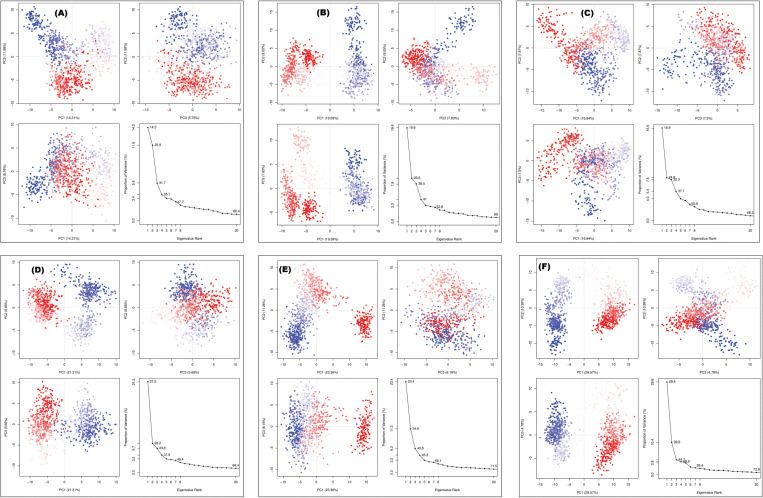
The Principal Component Analysis (PCA) plotted against the percentage of variance. **(A)** Benzimidine (Control), **(B)** CID: 72307 (sesamin), **(C)** CID: 5471610 (7-epiclusianone), **(D)** CID: 9984117 [(1S,4R,8S,10R)-8-benzoyl-4-(2-hydroxypropan-2-yl)-9,9-dimethyl-1,10-bis(3-methylbut-2-enyl)-3-oxatricyclo[6.3.1.02,6]dodec-2(6)-ene-7,12-dione)], **(E)** CID: 10791588 [(1S,9S,11R)-9-benzoyl-4,4,10,10-tetramethyl-1,11-bis(3-methylbut-2-enyl)-3-oxatricyclo[7.3.1.02,7]tridec-2(7)-ene-8,13-dione)], **(F)** CID: 129827386 [(s)-(+)-3-(3,4-Dihydroxy-phenyl)-acrylic acid 2,2-dimethyl-8-oxo-3,4-dihydro-2h, 8h-pyrano [3,2-g]-chromen-3-yl-ester)].

Principal component analysis (PCA) is a powerful statistical technique used to simplify complex datasets by identifying patterns and correlations among variables. In this study, PCA was employed to analyze the mobility of the protein backbone throughout MD simulations [69]. By decomposing the trajectory data into principal components (PCs), which represent the major axes of variation, we characterized the dynamic behavior of the protein and assessed the effects of ligand binding on its conformational dynamics. The mobility of the protein backbone based on the MD trajectories, was primarily explained by the first three principal components (PCAs), as shown in **Fig 7**. The PCA eigenvalue was plotted against the percentage of variance, revealing six distinct sections of variation. The relatively low variation percentages for the control suggest that additional factors, beyond the first three principal components, contribute to the overall conformational dynamics of the protein (Fig 7A**).** Comparing the variance percentages of different ligand-bound complexes provides insights into how ligand binding influences the protein’s conformational flexibility. Differences in variance percentages across ligands suggest that each ligand induces distinct conformational changes in the protein backbone, possibly due to variations in binding energy, interaction mode, or allosteric effects. Ligands 72307 and 5471610 exhibited low variations in PC1, PC2, and PC3 (Fig 7B **and** 7C). In contrast, ligands 10791588 and 9984117 showed notable variance in PC1, suggesting significant effects on the protein’s conformational dynamics upon binding. However, differences in the contributions of PC2 and PC3 imply distinct modes of interaction or structural rearrangements induced by these ligands (**Fig 7D and 7E**). Ligand 129827386 displayed a higher percentage of variance for PC1, indicating greater variability in protein backbone mobility compared to other ligands. This suggests that ligand 129827386 induces pronounced conformational changes in the protein structure, potentially affecting its function or stability (**Fig 7F**).

### 3.5. MM-GBSA binding free energy calculations

The most stable compound was identified using the MM-GBSA technique, which calculates the binding free energies of each complex. The MM-GBSA approach provides a more precise method than molecular docking for determining the binding energy of a ligand at a protein’s active site. It measures the energy difference between the energetically optimized ligand-receptor complex and the optimized states of the free ligand and receptor [70,71]. Following the MD simulation experiments, each nanosecond was divided into 1000 frames (0.1, 0.2, 0.3, 0.4, 0.5, 0.6, 0.7, 0.8, 0.9, and 1) for detailed analysis. The ΔG binding values (ΔG Bind) of the MT-SP1 protein complexed with the co-crystal (control) and ligands 72307, 5471610, 9984117, 10791588, and 129827386 were calculated as 16.18, -48.933, -39.641, -50.160, -37.322, and -67.600 kcal/mol, respectively. Based on MM-GBSA ΔG binding calculations, ligands 129827386, 9984117, and 72307 were identified as the most promising propolis-derived compounds for inhibiting MT-SP1, demonstrating strong energetic interactions with the target protein (MT-SP1) (**Table 2).** Considering MM-GBSA ΔG binding energy calculations, MD simulations (RMSD, RMSF, and protein-ligand contacts), and molecular docking studies (binding energy and protein-ligand contacts), all five propolis-derived small compounds were identified as potential inhibitors of the MT-SP1 protein. Therefore, these compounds should be further studied to validate their drug-likeness and therapeutic potential.

**Table 2:** MM-GBSA binding free energies of the MT-SP1-ligand complexes.

MT-SP1-ligand complex	MM-GBSA ΔG Bind (kcal/mol)	MM-GBSA ∆G Bind Coulomb (kcal/mol)	MM-GBSA ∆G Bind Covalent (kcal/mol)	MM-GBSA ∆G Bind H bond (kcal/mol)	MM-GBSA ∆G Bind Lipo (kcal/mol)	MM-GBSA ∆G Bind Solv GB (kcal/mol)	MM-GBSA ∆G Bind vdW (kcal/mol)
MT-SP1 (co-crystal)	16.189	–11.312	0.840	–3.171	–5.698	15.944	–23.844
MT-SP1–72307	–48.933	–11.214	1.401	–0.057	–21.045	21.439	–36.924
MT-SP1–5471610	–39.641	34.801	–0.288	–0.799	–15.473	–17.971	–39.910
MT-SP1–9984117	–50.160	–8.582	0.194	–0.689	–14.086	22.301	–49.287
MT-SP1–10791588	–37.322	–10.305	1.956	–0.466	–16.415	31.661	–43.753
MT-SP1–129827386	–67.600	–10.332	2.178	–0.830	–22.327	16.311	–46.363

### 3.6. Density functional theory (DFT) studies of MT-SP1-propolis-derived small molecules complex

**Fig 8:**
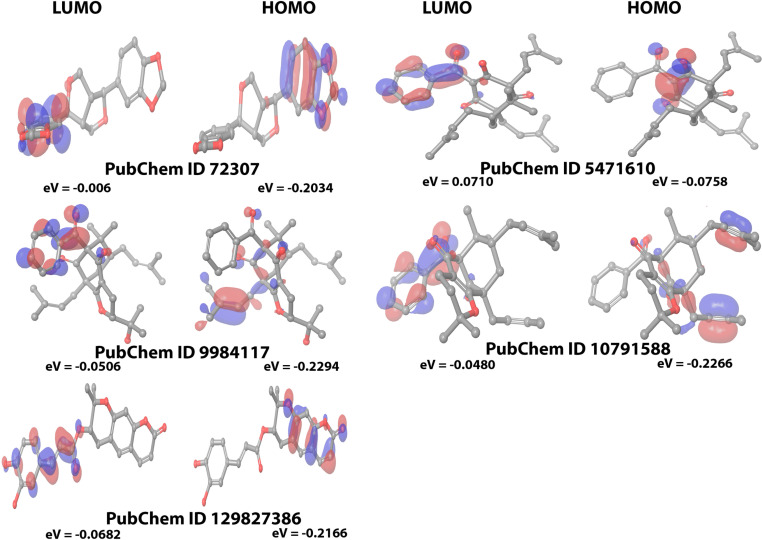
The LUMO and HOMO orbitals of propolis derived small molecules. The colors of the atoms are carbon (grey), hydrogen (white), and oxygen (red); the colors of the contour surface are blue (electron depletion) and red (negative electron density), which shows where electrons are most likely to be observed or found.

In this study, DFT (Density Functional Theory) analysis provided valuable insights into the interaction between propolis-derived small molecules and the MT-SP1 protein, highlighting their therapeutic potential as inhibitors of breast cancer proliferation and metastasis. DFT analysis was employed to investigate the electrical characteristics and binding energy patterns of the top-ranked MT-SP1–propolis-derived small-molecule complexes. The electronic structures and properties of the selected compounds were described using the 6–311 G basis set and the B3LYP exchange-correlation functional, using Koopman’s theorem, to analyze molecular orbitals [72]. The DFT parameters for all selected propolis-derived small molecules are listed in **Table 3**. At the MT-SP1 active site, the chosen ligands exhibited strong reactivity and chemical stability, as indicated by the minimal energy gap between the HOMO and LUMO **Fig 8** [73]. The electronegativity (χ) values of all selected ligands suggested favorable interactions with the MT-SP1 protein, leading to strong electrostatic interactions, which are critical for effective inhibition. The positive electronic chemical potential (μ) values indicated spontaneous chemical reactivity, suggesting that these compounds could undergo chemical transformations within the protein environment, contributing to their inhibitory activity against MT-SP1. The positive chemical hardness (η) and chemical softness (S) values for the selected compounds indicate a balanced combination of stability and flexibility, allowing effective interactions with the protein target. Additionally, the positive polarizability (σ) and electrophilicity (ω) values suggest that these ligands can undergo induced dipole interactions with their surrounding environment. This adaptability enhances their binding energy, potentially improving their inhibitory effects. Overall, DFT analysis confirmed that propolis-derived small molecules demonstrated stable interactions with the MT-SP1 protein, which is crucial for developing effective inhibitors due to structural integrity and inhibitory activity of these ligands.

**Table 3:** DFT parameters of top-ranked propolis-derived small compounds.

Propolis-derived small compounds	LUMO	HOMO	ΔE in eV	χ Pauling	η in eV	σ	μ in eV	S	ω eV
72307	-0.00623	-0.2034	0.197182	-0.10482	0.09859	10.1429	0.1048	5.0714	0.05572
5471610	0.071083	-0.07584	0.146925	-0.00238	0.07346	13.6123	0.0023	6.806	3.86
9984117	-0.05065	-0.22949	0.178845	-0.14008	0.08942	11.18283	0.1400	5.5914	0.1097
10791588	-0.04806	-0.22662	0.178557	-0.13735	0.08927	11.20089	0.1373	5.6004	0.1056
129827386	-0.06825	-0.21664	0.148390	-0.14245	0.07419	13.47793	0.1424	6.7389	0.1367

To contextualize our computational findings, we compared the binding affinities and electronic properties of our top five propolis-derived compounds with previously reported matriptase inhibitors. Oyebamiji et al. (2024) evaluated nine optimized heterocyclic active compounds and reported binding energies ranging from –7.79 to –8.80 kcal/mol [72]. In contrast, our study revealed that propolis-derived molecules exhibited even stronger interactions, as evidenced by their significant binding energies (Table 1). Furthermore, HOMO-LUMO analysis showed that our selected compounds had a smaller energy gap (ΔE) compared to previously studied inhibitors, indicating enhanced electronic reactivity. While this feature suggests strong inhibited potential, it may also indicate reduced stability under physiological conditions. Therefore, further validation through *in vitro* and *in vivo* studies is essential to assess the therapeutic viability of these compounds.

To support these computational predictions, enzymatic and cell-based activity assays of MT-SP1 can conducted to confirm the inhibitory potential of the identified propolis compounds. This assessment would involve measuring the reduction in signal resulting from the cleavage of a fluorophore-labeled synthetic peptide substrate by activated MT-SP1 [74]. Additionally, a fluorescence-based protease activity assay using conditioned media from treated and untreated MCF-7 breast cancer cell lines could determine whether these compounds interfere with the activation of matriptase from its zymogenic state [75].

### 3.7. Computational prediction related to drug-likeness, pharmacokinetics screening, and toxicity analysis of selected propolis-derived small molecules

**Fig 9:**
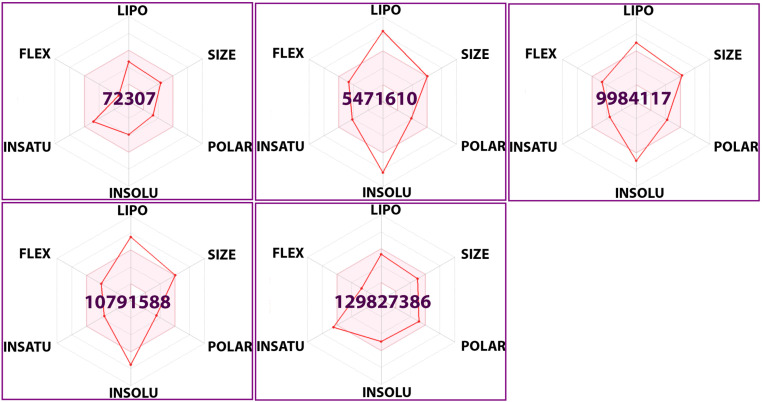
The oral bioavailability radar for physicochemical analysis of selected top-ranked propolis-derived small molecules. In the bioavailability radar, the colored-zone represents the appropriate physicochemical space for oral bioavailability. The reference ranges include LIPO (lipophilicity): -0.7 <XLOGP3 <+5.0); SIZE: 150 g/mol <MW < 500 g/mol; POLAR (Polarity): 20 Å² <TPSA < 130 Å²; INSOLU (Insolubility): -6 <LogS (ESOL) < 0; INSATU (Instauration): 0.25 <Fraction Csp3 < 1; FLEX (Flexibility): 0 <Number of rotatable bonds < 9.

Computer-aided drug-likeness evaluation, pharmacokinetic screening, and toxicity analysis are essential for selecting lead compounds from a diverse set chemicals. These processes help identify compounds with an optimal balance of efficacy and safety while enabling further optimization for eventual clinical use. By prioritizing compounds with the highest likelihood of success, these analyses minimize the risk of failure during drug development [58,76]. In this study, drug-likeness, pharmacokinetics screening, and toxicity analysis of selected top-ranked propolis-derived small molecules were performed using SwissADME, OSIRIS Property Explorer, and ADMET lab platforms. The molecular weights of the two compounds, ligands 72307 and 129827386, were below 500 g/mol, aligning with Lipinski’s Rule of Five and other drug-likeness criteria. Although the remaining three compounds slightly exceeded this molecular weight threshold, they still exhibited favorable drug-likeness properties, each with only one rule violation. Additionally, ligands 72307 and 129827386 had slightly higher numbers of hydrogen bond acceptors and donors compared to the other compounds. However, they attained the highest drug scores, indicating superior drug-like properties. Notably, all five ligands demonstrated a bioavailability score greater than 0.55, along with satisfactory solubility parameters—critical factors for effective absorption and distribution in the body. Toxicity screening revealed no significant toxicity risks for mutagenicity, tumorigenicity, irritancy, and/or reproductive risk effects in four of the five selected ligands. However, ligand 129827386 was associated with potential reproductive risk, warranting further investigation. Pharmacokinetic screening indicated favorable properties across all compounds, particularly for ligands 72307 and 129827386, which exhibited high gastrointestinal absorption. Moreover, the LD50 values of these two ligands fell within the low-toxicity range, and their half-life and clearance rates were acceptable, further supporting their potential as drug candidates. Based on these findings, ligands 72307 and 129827386 emerged as the most promising candidates for future drug design studies (**Table 4**). Additionally, bioavailability radar analysis of these propolis-derived small molecules validated these results (**Fig 9**). The bioavailability radar for ligand 72307 confirmed that all its relevant properties were within the acceptable range, while five out of six properties for ligand 129827386 remained within favorable limits, further reinforcing their potential for drug development.

**Table 4:** Computation of drug-likeness, toxicity, and pharmacokinetics analysis of selected propolis-derived chemical compounds.

Properties	Compounds PubChem ID				
**72307**	**129827386**	**9984117**	**5471610**	**10791588**
**Drug Likeness screening**					
Molecular weight	354	408	518	502	502
No. H-bond acceptors	6	7	5	4	4
No. H-bond donor	0	2	1	1	0
No. of rotational Bond	2	4	7	7	6
Topological polar surface area (TPSA - Å²)	55.38	106.2	80.67	71.44	60.44
Lipinski rule	Yes	Yes	Yes; 1 violation: MW>500	Yes; 1 violation: MW>500	Yes; 1 violation: MW>500
Ghose rule	Yes	Yes	No	No	No
Veber’s rule	Yes	Yes	Yes	Yes	Yes
Muegge’s rule	Yes	Yes	No	No	No
Egan’s rule	Yes	Yes	No	No	No
log*S*	0.65	0.63	0.24	0.336	0.176
*c*Log*P*	3.22	3.4	6.61	8.24	7.44
Solubility	–4.39	–4.45	–6.13	–5.68	–6.54
Drug likeness	0.083	–2.7	–0.42	–0.09	–2.79
Drug score	0.54	0.21	0.18	0.19	0.13
Bioavailability Score	0.55	0.55	0.56	0.85	0.85
**Toxicity Risk**					
Mutagenicity score^a^	1	1	1	1	1
Tumorigenicity score^a^	1	1	1	1	1
Irritating effect score^a^	1	1	1	1	1
Reproductive effect score^a^	1	0.6	1	1	1
**Pharmacokinetics Screening**					
Gastrointestinal absorption	High	High	Low	Low	Low
BBB permeation	Yes	No	No	No	No
P-glycoprotein substrate	No	No	Yes	Yes	Yes
CYP1A2 inhibitor	No	No	No	No	No
CYP2C19 inhibitor	Yes	No	No	No	No
CYP2C9 inhibitor	No	Yes	No	No	No
CYP2D6 inhibitor	Yes	No	No	No	No
CYP3A4 inhibitor	Yes	No	Yes	Yes	Yes
T _1/2_ (Half-Life Time - hours)/ Clearance Rate (Cl: mL/min/kg)	1.997/ 1.871	1.749/ 1.51	2.184/ 1.616	2.184/ 1.465	2.112/ 1.401
Log Kp (skin permeation)	–6.56 cm/s	–6.05 cm/s	–4.70 cm/s	–2.89 cm/s	–3.91 cm/s
LD_50_	559.039 mg/kg	543.366 mg/kg	131.799 mg/kg	423.94 mg/kg	184.631 mg/kg

***Abbreviations with reference range:*** MW: molecular weight, HA: number of heavy atoms, ARO HA: number of aromatic heavy atoms, FCsp³: fraction of carbons in the sp³ hybridization (≥ 0.25), RB: rotatable bond (≤ 10), HBA: hydrogen bond acceptor (≤ 10), HBD: hydrogen bond donor (≤ 5), MR: molar refractivity (≤ 130), TPSA: topological polar surface area (≤ 150), a = risk score (No risk = 1, moderate risk = 0.8 and high risk = 0.6), BBB: Blood Brain Barrier, CYP: Cytochrome, T _1/2_ (Half-Life Time - hours) = Range:>8h: high; 3h<Cl <8h: moderate; <3h: low, Range: >15 mL/min/kg: high; 5mL/min/kg<Cl <15mL/min/kg: moderate; <5 mL/min/kg: low, LD50 (LD50 of acute toxicity): High-toxicity: 1~50 mg/kg; Toxicity: 51~500 mg/kg; low-toxicity: 501~5000 mg/kg.

## 4. Conclusion

This study employed various computational methods to evaluate the potential inhibitory effects of propolis-derived compounds on breast cancer metastasis. Molecular docking and MD simulations were performed to analyze the binding interactions and stability of five propolis derivatives (ligands 72307, 5471610, 9984117, 10791588, and 129827386) with the MT-SP1 protein. The results showed that all top-ranked ligands exhibited strong binding energy towards MT-SP1, with ligands 72307, 9984117, 129827386, and 10791588 demonstrating enhanced stability and minimal conformational changes. PCA analysis provided insight into ligand dynamics within the protein-ligand complexes. Binding free energy calculations further confirmed the strong interactions between the top-ranked ligands and MT-SP1. Additionally, DFT analysis indicated that these propolis-derived small molecules maintained structural integrity and inhibitory activity, reinforcing their potential as MT-SP1 inhibitors. Pharmacokinetic analysis revealed that ligands 72307 and 129827386 exhibited high bioavailability, good solubility, low toxicity, and favorable half-life and clearance rates, making them promising drug candidates. Overall, these findings provide valuable molecular insights into the interactions between propolis derivatives and MT-SP1, laying the foundation for future experimental research and drug development for the treatment of metastatic breast cancer.

## Future recommendations

To further investigate propolis-derived compounds as potential breast cancer therapeutics, it is crucial to conduct cellular assays and utilize animal models of breast cancer. These studies will help evaluate the efficacy, safety, and impacts of these compounds on tumor growth suppression, metastasis, and systemic toxicity. To enhance the bioavailability of these compounds, we proposed the use of nanocarriers and prodrug formulations of the active ingredients. Additionally, ligand receptor-mediated targeting and selectivity engineering – achieved through structural modifications of the selected compounds—can improve their binding specificity for matriptase, minimizing off-target effects.

## Supporting information

S1 Table Propolis-derived small molecules’ energetic and molecular interactions with MT-SP1 protein. (XLSX)
